# A standardized dataset for conservation prioritization of songbirds to support CITES

**DOI:** 10.1016/j.dib.2021.107093

**Published:** 2021-05-07

**Authors:** Jacqueline Juergens, Simon Bruslund, Johanna Staerk, Rikke Oegelund Nielsen, Chris R. Shepherd, Boyd Leupen, Kanitha Krishnasamy, Serene Chui Ling Chng, John Jackson, Rita da Silva, Antony Bagott, Romulo Romeu Nóbrega Alves, Dalia A. Conde

**Affiliations:** a Biological Faculty, University of Hamburg, Martin-Luther-King-Platz 3, 20146 Hamburg, Germany; b Department of Biology, University of Southern Denmark, Campusvej 55, 5230 Odense M, Denmark; c Species360 Conservation Science Alliance, 7900 International Drive, Suite 1040, Bloomington, MN 55425, USA; d Bird Park Marlow, Kölzower Chaussee 1, 18337 Marlow, Germany; e European Association of Zoos and Aquaria - Songbird Taxon Advisory Group and Silent Forest Group, c/o Artis Zoo - PO Box 20164, 1000 HD Amsterdam, The Netherland; f Interdisciplinary Centre on Population Dynamics, University of Southern Denmark, 5230 Odense M, Denmark; g Department of Mathematics and Computer Science, University of Southern Denmark, 5230 Odense M, Denmark; h Monitor Conservation Research Society (Monitor), Box 200, Big Lake Ranch, B.C., V0L 1G0, Canada; i TRAFFIC International - Southeast Asia, Suite 12A-01, Level 12A, Tower 1, Wisma AmFirst, Jalan Stadium SS 7/15, 47301 Kelana Jaya, Selangor, Malaysia; j TRAFFIC, David Attenborough Building, Pembroke Street, Cambridge, CB2 3QZ, UK; k Programa de Pós-graduação em Etnobiologia e Conservação da Natureza, Departamento de Biologia, Universidade Estadual da Paraíba, Av. das Baraúnas, 351 / Campus Universitário, Campina Grande, PB, 58109-753, Brazil

**Keywords:** Wildlife, Trade, Species management, International conventions, Threatened species, Migratory species, Life history traits, Ex-situ

## Abstract

In this article we present a standardized dataset on 6659 songbirds (Passeriformes) highlighting information relevant to species conservation prioritization with a main focus to support the Convention on International Trade in Endangered Species of Wild Fauna and Flora (CITES). Data were collected from both scientific and grey literature as well as several online databases. The data are structured into six knowledge categories: Conventions and Treaties, Human Use, Extinction Risk, Management Opportunities, Biological Information, and Intrinsic Values. The Conventions and Treaties category includes the listings for two international conventions, CITES and the Convention on the Conservation of Migratory Species of Wild Animals (CMS), as well as EU listings for the EU Wildlife Trade Regulations and the EU Birds Directive. The Human Use category contains information on both regulated trade collected from the CITES Trade Database and the United States’ Law Enforcement Management Information System (LEMIS), and highly aggregated data on seizures which we obtained from TRAFFIC, the United Nations Office on Drugs and Crime (UNODC) and two data sources on traditional medicine. We also present, for the first time, the complete Songbirds in Trade Database (SiTDB), a trade database curated by taxon expert S. Bruslund based on expert knowledge, literature review, market surveys and sale announcements. Data on the types of human use, including traditional medicine are also provided. The knowledge area on Extinction Risk contains data on the species’ IUCN Red List status, the Alliance for Zero Extinction Trigger Species status, site and population at the site, the species’ IUCN Climate Change Vulnerability Assessment, and the listing of priority species at the Asian Songbird Crisis Summit. In the Management Opportunities category, we gathered data on ex-situ management from Species360 zoo holdings as well as species management plans from the European and North American Zoo Associations (EAZA and AZA, respectively). Biological Information includes data on body mass, clutch size, diet, availability of data from the IUCN Red List on habitat systems, extent of occurrence, generation length, migration pattern, distribution, and biological data from the Demographic Species Knowledge Index, number of occurrences recorded by the Global Biodiversity Information Facility (GBIF) as well as genomic data from the Bird 10 000K Genomes (B10K) project, Vertebrate Genome Project (VGP) and GenBank. Information on invasive species is also part of this knowledge area. The Intrinsic Value category refers to two measures of the species’ intrinsic value, namely Ecological and Evolutionary Distinctiveness. In order to make these knowledge areas comparable, we standardized data following the taxonomy of the Handbook of the Birds of the World and Birdlife (Version 4, 2019). The data enable a broad spectrum of analyses and will be useful to scientists for further research and to policymakers, zoos and other conservation stakeholders for future prioritization decisions.

## Specifications Table

**Table:** 

Subject	Species Conservation, Management, Monitoring, Policy, Law
Specific subject area	Biology, Aves, Passeriformes, IUCN Red List, Vulnerability to Climate Change, CITES, CMS, AZE, EDGE, Captive husbandry, Genomics, Wildlife trade, Life history traits
Type of data	Table Chart Figure
How data were acquired	Data: Online databases, scientific and grey literature, webpages Software: R (R Core Team, 2020)
Data format	Raw Analysed Filtered
Parameters for data collection	Data were collected across the knowledge areas of Conventions and Treaties, Human Use, Extinction Risk, Management Opportunities, Biological Information and Intrinsic Values under the Species Knowledge Index methodology (Conde et al. 2019) for the 6659 Passeriformes species described by the Handbook of the Birds of the world and Birdlife (Version 4, 2019). In addition, we added data on songbird trade from the grey literature, publications, websites, and expert visits to markets to existing information in the Songbirds in Trade Database (SiTDB). The aims were to collect and standardize data relevant for the prioritization of species conservation actions to support the decision-making process that regulates species’ international trade by CITES, and to identify knowledge gaps for future research.
Description of data collection	Data were collected from open online databases, websites, and supplementary data from peer-reviewed publications. The links to all publicly available data are provided in Table 1. Data on zoo species holdings in the ZIMS database were provided directly by Species360. All data compiled on songbird trade from grey literature, social media sales postings, market visits by experts, and expert opinion were compiled and standardized into a unique spreadsheet that we named The Songbirds in Trade Database (SiTDB), curated and led by S. Bruslund. Species Survival plans from the American Association from Zoos and Aquariums (AZA) were obtained from private communication with M. Brauns. Taxonomy was standardized following the Handbook of the Birds of the World and Birdlife taxonomy (Version 4, 2019). All data processing and analyses were carried out using the open-source software R.
Data source location	Global data and regional data for trade and ex-situ programs (Europe, North America & Global), legislation (Europe & Global) and traditional medicine use (Africa & Global)
Data accessibility	With the article, in Dryad (https://datadryad.org/stash/share/HuesuylSEF0xqoY96j7twjggZt54A8O474ZQnoxetRc), and in the Species360 Open Data Repository

## Value of the Data

• The data provided here will be useful to support the decision-making process by the Convention on International Trade in Endangered Species of Wild Fauna and Flora (CITES), in particular for the implementation of ***Decision, 18.256 on Songbird trade and conservation management***, taken at the 18th meeting of the CITES Conference of the Parties, Geneva, 2019. It is an integral part of the Species Knowledge Initiative to Support CITES Decisions and Recommendations for Songbirds [1].
• The data are useful for conservation practitioners and policy makers to identify both species of conservation priority and opportunities for protection in the highly diverse songbird group. Therefore, these data can inform decision making for the development of legislative measures as well as ex-situ and in-situ species management programs.
• The data we present can be used by researchers for the development of comparative analyses across the songbirds, given the comprehensive standardized dataset for 6659 songbird species containing information on species life-history traits and 4368 taxonomic synonyms across the 32 data repositories used here.
• The data we provide support decision making on future project endeavours by highlighting knowledge gaps and opportunities for the advancement of songbird research.

## 1 Data Description

This dataset contains species level information on the 6659 songbird species (Order Passeriformes) described by the Handbook of the Birds of the World and BirdLife International (Version 4, 2019)(HBW/Birdlife) [2], including the 60 extinct species in their list. The data were collated from 32 sources including scientific and grey literature, websites and online databases and cover six knowledge categories: Convention and Treaties, Human Use including regulated trade and seizures, Extinction Risk, Management Opportunities, Biological Information and Intrinsic Values. The entire dataset is provided in Supplementary File S1. Metadata i.e. data and variable descriptions as well as sources are available in a separate data sheet in Supplementary File S2. Table 1 shows the number of species covered by each data source. To make the datasets comparable, we standardized the taxonomy according to HBW/Birdlife using a synonym list (for details see Experimental Design, Materials and Methods). The complete synonym list used for standardization is provided in Supplementary File S3.

**Table 1:** Data repositories and sources used for the taxonomic standardization and across the six knowledge areas. Here we give the number of passerine species for which data was collected from each dataset, the number of species that could be matched to the taxonomy of the Handbook of the World and Birdlife (HBW/Birdlife), and the dataset sources and links if available. Discrepancies between the number of species is due to taxonomic differences and/or other data cleaning steps (see Experimental Design, Materials and Methods). The original taxonomic authority used for each dataset is also given when reported. Note that some databases contain data used in more than one knowledge category. However, for simplicity we only list the database in one of the knowledge categories (refer to the source column in the supplementary data for more detail). CoL = Catalogue of Life, HBW/Birdlife = Handbook of the Birds of the world and BirdLife, TAS = The Howard and Moore Complete Checklist of the Birds of the World.

Dataset title	Number of species	Number of BirdLife species	Access	Original Taxonomy	Reference	Source
Taxonomy						

Handbook of the Birds of the World and BirdLife International digital checklist of the birds of the world. Version 4.	6659	6659	April 2020	HBW/Birdlife	Handbook of the Birds of the World and BirdLife International, Handbook of the Birds of the World and BirdLife International digital checklist of the birds of the world. Version 4., (2019).	http://datazone.birdlife.org/home http://datazone.birdlife.org/species/taxonomy

ITIS Passeriformes Report	6264	6119	March 2020		ITIS, Integrated Taxonomic Information System on-line database, (2020). https://www.itis.gov.	https://www.itis.gov/servlet/SingleRpt/SingleRpt?search_topic=TSN&anchorLocation=SubordinateTaxa&credibilitySort=Subordinate%20Taxa&rankName=Species&search_value=178265&print_version=SCR&source=from_print#SubordinateTaxa

Avibase Handbook of the Birds of the World and BirdLife Synonyms			February - December 2020	HBW/Birdlife	D. Lepage, Avibase - The World Bird Database, (2020). D. Lepage, private communication	https://avibase.bsc-eoc.org/avibase.jsp?lang=EN

**1.1. Conventions and Treaties**						

Convention on International Trade in Endangered Species of Wild Fauna and Flora (CITES)	85	93	April 2020	TAS	UNEP, The Species+ Website, Nairobi, Kenya. Compiled by UNEP-WCMC, Cambridge, UK, (2020).	https://www.speciesplus.net/

History of CITES Listings	110	110	November 2020	TAS	UNEP-WCMC (Comps.), Checklist of CITES species, Hist. CITES List. (2014)	http://checklist.cites.org/

The Convention on the Conservation of Migratory Species of Wild Animals (CMS)	442	432	February 2020	HBW/Birdlife	UNEP, The Species+ Website, Nairobi, Kenya. Compiled by UNEP-WCMC, Cambridge, UK, (2020).	https://www.speciesplus.net/
European Union Wildlife Trade Regulations	127	126	September 2020	HBW/Birdlife	UNEP, The Species+ Website, Nairobi, Kenya. Compiled by UNEP-WCMC, Cambridge, UK, (2020).	https://www.speciesplus.net/

List of birds of the European Union	53	53	December 2020	HBW/Birdlife	Council Directive 2009/147/EC on the conservation of wild birds, Official Journal L 020, p. 7, (2009).	https://ec.europa.eu/environment/nature/conservation/wildbirds/eu_species/index_en.htm

**1.2. Human Use**						

Trade						

CITES Trade Database	222	177	June 2020	TAS	UNEP World Conservation Monitoring Centre, CITES trade statistics derived from the CITES Trade Database, Cambridge, UK. (2020).	https://trade.cites.org/

IUCN Advanced Search, Usetrade	2138	2138	June 2020	HBW/Birdlife	IUCN, IUCN Red List of Threatened Species, Version 2019-1. (2019).	https://www.iucnredlist.org/

Songbirds in Trade Database (SiTDB)	6660	6659	September 2020	HBW/Birdlife	Songbirds in Trade database	This paper

United States Fish and Wildlife Service's (USFWS) Law Enforcement Management Information System (LEMIS)	417	286	-		E.A. Eskew, A.M. White, N. Ross, K.M. Smith, K.F. Smith, J.P. Rodríguez, C. Zambrana-torrelio, W.B. Karesh, P. Daszak, United States wildlife and wildlife product imports from 2000 – 2014, (2020) 1–8.	https://doi.org/10.1038/s41597-020-0354-5.

TRAFFIC Wildlife Trade Information System (WiTIS)	371	259	September 2020		TRAFFIC, Passerine Incidents 2008-2020, Incident dataset, 2020	Private communication

World WISE Database	73	70	November 2020		UNODC, World WISE Database, List of Songbirds Records, (2020).	Private communication

Traditional Medicine						

CITES List of species use in Traditional Medicine	3	3	January 2020	TAS	CITES, AC18 Doc. 13.1., List of species traded for medicinal purposes., (2002).	https://cites.org/sites/default/files/eng/com/ac/18/E18-13-1.pdf
Birds of a Feather: Quantitative Assessments of the Diversity and Levels of Threat to Birds Used in African Traditional Medicine	106	106	January 2020	HBW/Birdlife	V.L. Williams, A.B. Cunningham, R.K. Bruyns, A. Kemp, Birds of a Feather: Quantitative Assessments of the Diversity and Levels of Threat to Birds Used in African Traditional Medicine, in: R. Alves, I. Rosa (Eds.), Anim. Tradit. Folk Med., Springer, Heidelberg, 2013: pp. 383–420.	https://doi.org/10.1007/978-3-642-29026-8_18

**1.3. Extinction Risk**						

2018 Global AZE map	102	102	April 28, 2020		Alliance for Zero Extinction, 2018 Global AZE Map, (2020). https://zeroextinction.org/	https://zeroextinction.org/site-identification/2018-global-aze-map/

Identifying the World's Most Climate Change Vulnerable Species: A Systematic Trait-Based Assessment of all Birds, Amphibians and Corals	5847	5782			W.B. Foden, S.H.M. Butchart, S.N. Stuart, J.C. Vié, H.R. Akçakaya, A. Angulo, L.M. DeVantier, A. Gutsche, E. Turak, L. Cao, S.D. Donner, V. Katariya, R. Bernard, R.A. Holland, A.F. Hughes, S.E. O'Hanlon, S.T. Garnett, Ç.H. Şekercioǧlu, G.M. Mace, Identifying the World's Most Climate Change Vulnerable Species: A Systematic Trait-Based Assessment of all Birds, Amphibians and Corals, PLoS One. 8 (2013). https://doi.org/10.1371/journal.pone.0065427.	https://doi.org/10.1371/journal.pone.0065427

IUCN Red List of Threatened Species Status	6659	6659	April 2020	HBW/Birdlife	Handbook of the Birds of the World and BirdLife International, Handbook of the Birds of the World and BirdLife International digital checklist of the birds of the world. Version 4., (2019).	http://datazone.birdlife.org/userfiles/file/Species/Taxonomy/HBW-BirdLife_Checklist_v4_Dec19.zip.

Asian Songbird Crisis Summit Priority Species	28	28	Sept 2020	HBW/Birdlife	J.G.H. Lee, S.C.L. Chng, J.A. Eaton, Conservation Strategy for Southeast Asian Songbirds in Trade, 2016.	https://doi.org/10.13140/RG.2.2.12805.96483.
**1.4. Management opportunities**						

Species360 Zoological Management System	2018	1910	February 2020		Species360, Zoological Information Management System (ZIMS), (2020).	https://zims.Species360.org

EAZA Passerine Taxon Advisory Group Regional Collection Plan for Songbirds. First Edition	175	175	February 2020		D. Jeggo, T. Pagel, EAZA Passerine Taxon Advisory Group Regional Collection Plan for Songbirds, in: S. Bruslund (Ed.), 1st ed., Cologne & Heidelberg, 2018: Table 6, pp. 6 - 11. General explanation in: D. Jeggo, T. Pagel, Passeriformes, EAZA, TAG Reports, 2017: p. 26 - 28	General explanation: https://www.eaza.net/assets/Uploads/Annual-report/1035-TAG-reports-2017-web.pdf

Regional Collection Plan of the EAZA Passeriformes Taxon Advisory Group, Asian Songbirds – Edition One.	135	130	February 2020		D. Jeggo, S. Bruslund, K. Traylor-Holzer, W. Van Lint, R. Van der Meer, Regional Collection Plan of the EAZA Passeriformes Taxon Advisory Group, Asian Songbirds – Edition One., 2019: Table 2, 8 – 17 pp.	Internal publication

AZA Species Survival Plans	31	31			M. Brauns, Pers. Communication	

**1.5. Biological information**						

Body Mass Median/Litter Clutch Size/ Diet	10254	5850/2911/5769	March 20	HBW/Birdlife	R.S.C. Cooke, A.E. Bates, F. Eigenbrod, Global trade-offs of functional redundancy and functional dispersion for birds and mammals, Glob. Ecol. Biogeogr. 28 (2019) 484–495.	https://doi.org/10.6084/m9.figshare.5616424.v1

Vertebrate Genome Project Database - VGP Phase I Genomes	3	3	March 20		K.-P. Koepfli, B. Paten, S.J. O'Brien, The Genome 10K Community of Scientists, The Genome 10K Project: A Way Forward, Annu. Rev. Anim. Biosci. 3 (2015) 57–111. 10.1146/annurev-animal-090414-014900.	http://vgpdb.snu.ac.kr/details/

Bird 10 000 Genomes (B10K) Project - Passeriformes	1363	962	April 20		G. Zhang, Bird sequencing project takes off, Nature. 52 (2015). https://doi.org/10.1038/522034d.	https://b10k.genomics.cn/species.html
GenBank	5060	4990			D.A. Benson, M. Cavanaugh, K. Clark, I. Karsch-Mizrachi, D.J. Lipman, J. Ostell, E.W. Sayers, GenBank, Nucleic Acids Res. D1 (2017) D37–D42. https://doi.org/10.1093/nar/gkw1070.	https://www.ncbi.nlm.nih.gov/genbank/

IUCN Red List Advanced Search, all_other_fields	6659	6659	June 20	HBW/Birdlife	IUCN, IUCN Red List of Threatened Species, Version 2019-1. (2019).	https://www.iucnredlist.org/

Distribution	6659	6659	March 20	HBW/Birdlie	IUCN, IUCN Red List of Threatened Species, Version 2019-1. (2019).	https://www.iucnredlist.org/

Demographic Species Knowledge Index	6239	6095	January 20	CoL	D.A. Conde, J. Staerk, F. Colchero, R. da Silva, J. Schöley, H. Maria Baden, L. Jouvet, J.E. Fa, H. Syed, E. Jongejans, S. Meiri, J.M. Gaillard, S. Chamberlain, J. Wilcken, O.R. Jones, J.P. Dahlgren, U.K. Steiner, L.M. Bland, I. Gomez-Mestre, J.D. Lebreton, J.G. Vargas, N. Flesness, V. Canudas-Romo, R. Salguero-Gómez, O. Byers, T.B. Berg, A. Scheuerlein, S. Devillard, D.S. Schigel, O.A. Ryder, H.P. Possingham, A. Baudisch, J.W. Vaupel, Data gaps and opportunities for comparative and conservation biology, Proc. Natl. Acad. Sci. U. S. A. 116 (2019) 9658–9664.	https://www.pnas.org/content/116/19/9658

Global Register of Migratory Species (GROMS)	1049	980	March 20	Sibley Monroe	K. Riede, The Global Register of Migratory Species ­ Database, GIS Maps and Threat Analysis, Landwirtschaftsverlag, Münster, 2001.	http://groms.de/groms_neu/view/order_stat_patt_spanish.php?search_pattern=

Global Invasive Species Database	15	15	September 2020	HBW/Birdlife	Invasive Species Specialist Group ISSG, The Global Invasive Species Database, Version 2015.1. (2015). http://www.iucngisd.org/gisd/	http://www.iucngisd.org/gisd/search.php
Alien Species in the EU	141	140	October 2020	HBW/Birdlife	European Commission - Joint Research Centre, European Alien Species Information Network (EASIN), (2020). https://easin.jrc.ec.europa.eu/	https://easin.jrc.ec.europa.eu/spexplorer/search/searchpaged

IAS of Union Concern	2	2	October 2020	HBW/Birdlife	European Commission - Joint Research Centre, European Alien Species Information Network (EASIN), (2020). https://easin.jrc.ec.europa.eu/	https://easin.jrc.ec.europa.eu/spexplorer/search/searchpaged

Occurrence data all records / only observations	6773/6620	6114/6095	August 2020	CoL	GBIF, GBIF Occurrence Download, (2020). https://doi.org/10.15468/dl.gfykvj GBIF, GBIF Occurrence Download, (2020). https://doi.org/10.15468/dl.96fvtc	https://doi.org/10.15468/dl.gfykvj https://doi.org/10.15468/dl.96fvtc

**1.6. Intrinsic value**						

Ecological distinctiveness of birds and mammals at the global scale	6591	6588	February 2020	HBW/Birdlife	R.S.C. Cooke, F. Eigenbrod, A.E. Bates, Ecological distinctiveness of birds and mammals at the global scale, Glob. Ecol. Conserv. 22 (2020) e00970. https://doi.org/10.1016/j.gecco.2020.e00970.	https://doi.org/10.1016/j.gecco.2020.e00970

Evolutionary Distinctiveness Scores - Birds	6590	6588	February 2020	HBW/Birdlife	Zoological Society of London, EDGE of Existence, EDGE List Birds. (2019). https://www.edgeofexistence.org/edge-lists/	https://www.edgeofexistence.org/edge-lists/

EDGE Birds	246	246	February 2020	HBW/Birdlife	Zoological Society of London, EDGE of Existence, EDGE List Birds. (2019). https://www.edgeofexistence.org/edge-lists/	https://www.edgeofexistence.org/edge-lists/

### 1.1 Conventions and treaties

We collected data on the listing of species in two international conventions: The Convention on International Trade in Endangered Species of Wild Fauna and Flora (CITES) and the Convention on the Conservation of Migratory Species of Wild Animals (CMS). We include historical data for CITES listing changes since 1975. In addition, we include listings in two European Union structures, the EU Birds Directive and the EU Wildlife Trade Regulations. Table 2 shows the number of species covered by each convention.

**Table 2:** Number of species per appendix or scheme for the four conventions listed in this dataset, CITES, CMS, the EU Wildlife Trade Regulations and the EU Birds Directive.

Convention	Appendix/Scheme	Number of species
CITES	I	12
	II	77
	III	4

CMS	I	4
	I/II	11
	II	417
	Aquatic Warbler*	1
	Southern South American Grassland Birds*	8

EU Wildlife Trade Regulations	A	13
	B	69
	C	3
	D	41

EU Bird Directive	I	39
	IIb	12

⁎ Taxa under a Memorandum of Understanding (MOU). MOUs are agreements between parties to protect particular taxa in addition to listings of species in the two CMS appendixes.

### 1.2 Human use

**Fig. 1:**
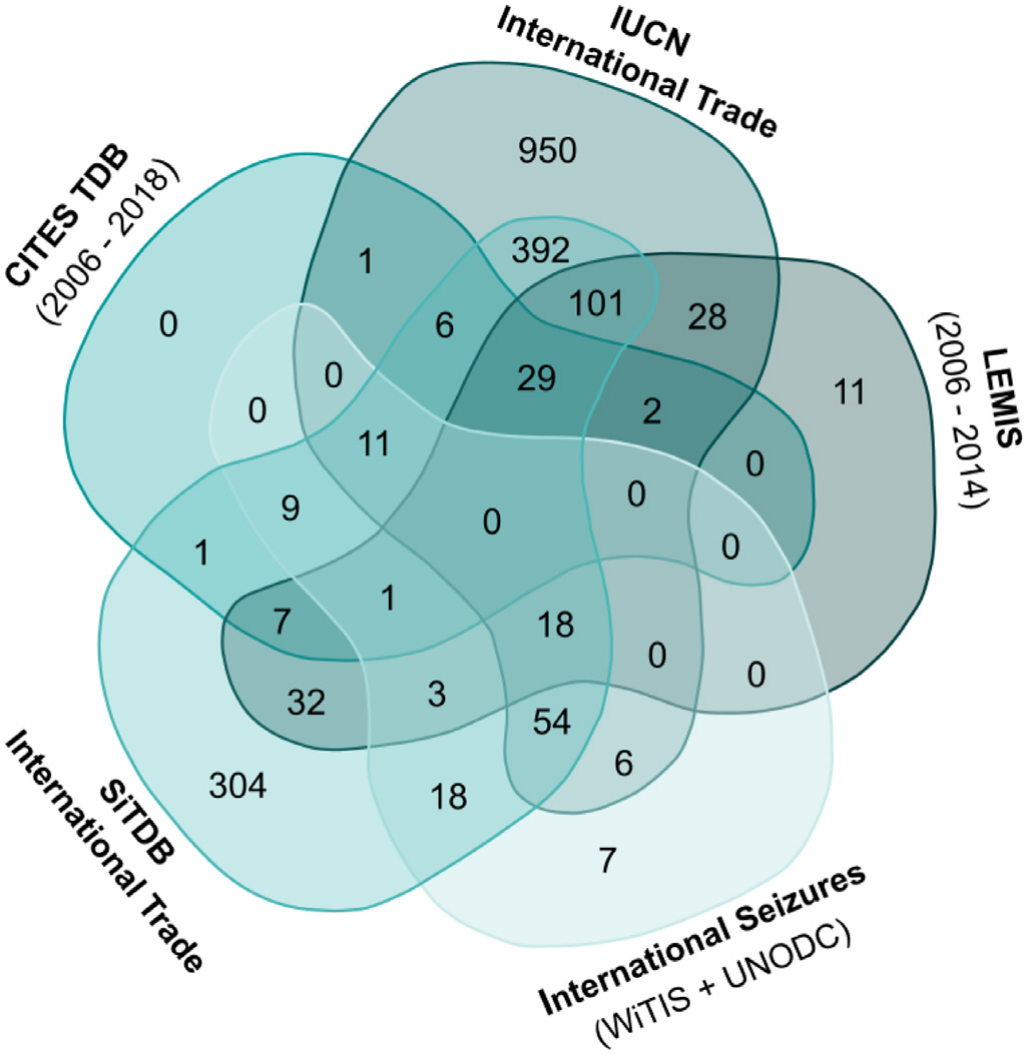
Venn diagram showing the number of species in each trade database and their overlap. To make the data sets comparable only data for live, commercially traded individuals since 2006 were used for the CITES Trade Database (CITES TDB) and the Law Enforcement Management Information System (LEMIS). For the TRAFFIC Wildlife Trade Information System (TRAFFIC International), the Songbirds in Trade Database (SiTDB International) and IUCN Red List (IUCN) only data referring to international trade entries were considered. This figure was generated using the Bioinformatics & Evolutionary Genomics webtool: (http://bioinformatics.psb.ugent.be/webtools/Venn/).

This category includes information on human use in the form of trade (international and domestic) and preliminary data on traditional medicine. We include data on regulated trade from the CITES Trade Database managed by the WCMC-UNEP, and the United States Fish and Wildlife Service's (USFWS) Law Enforcement Management Information System (LEMIS), data on illegal trade from the Wildlife Trade Information System (WiTIS) managed by TRAFFIC, the Songbirds in Trade Database (SiTDB), data on trade from the IUCN Red List, and data on traditional medicine from the *CITES List of species use in Traditional Medicine* and from a published quantitative assessment of birds used in African traditional medicine (Table 1). The number of species in international trade in each database and their overlap between databases is shown in Fig. 1.

#### 1.2.1 Regulated trade

Here we include information on international trade from the CITES Trade Database, as well as imports into the United States from the LEMIS database curated by the EcoHealth Alliance. Data from the CITES Trade Database contains trade information on CITES listed species and species listed by the European Union Wildlife Trade Regulations from 1975–2018. These data include annual import and export quantities, importing and exporting countries, as well as the origin country of the species, the purpose of trade (such as commercial, or hunting trophies), the source (such as captive-bred or wild-caught) and the term it was traded as (e.g live, feathers, bones etc.). We provide aggregated counts of the total number of live individuals traded for commercial purposes for the time period 2006–2018 per source (e.g. captive-bred, wild-caught). We provide both the importer reported quantities and the exporter reported quantities, which can show discrepancies. We further provide lists of the trade terms, sources and purposes per species in the years 2006–2018. The presence of a species in the CITES Trade Database for all sources and purposes from 1975–2018 and for live commercial trade separately are also reported here. We also downloaded data on wildlife and wildlife product imports into the United States from the LEMIS database. The data include the number of live individuals or individuals that died during transport traded for commercial purposes for each source (e.g., wild, captive-bred, unknown) as well as the countries and territories involved in the trade.

#### 1.2.2 Seizures

We obtained data on songbird seizures from the Wildlife Trade Information System (WiTIS) compiled by TRAFFIC and the World WISE database from the United Nations Office on Drugs and Crime (UNODC). The data from WiTIS include the number of individuals (alive or dead) that were seized in domestic or international trade as well as the number of incidents and involved countries between 2008 and 2020.

The UNODC kindly provided a list of species that were seized from 2006 to 2018, recorded in the UNODC World WISE database which we present here.

#### 1.2.3 Songbirds in trade database

We introduce the Songbirds in Trade Database (SiTDB) with the aim to provide information on live, commercial songbird trade between 2006 and 2020, that cannot be obtained from data sources such as the CITES Trade Database and TRAFFIC's WiTIS. Thus, we standardized and incorporated data from diverse sources ranging from peer-reviewed literature to social media and market visits. The SiTDB is led and curated by coauthor S. Bruslund and includes information on whether a species is traded domestically or internationally, trade interest (i.e. evidence of trade through observation, monitoring, surveys or trade adverts), the primary source of trade (wild caught vs. captive-bred), whether trade is a contributing threat to wild populations, the perceived trade volume relative to populations based on expert knowledge, information on ex-situ management, the difficulty of captive breeding, trade routes, affected subspecies, and an indication of domestication effect (for detailed explanations on each of these variables, see Experimental Design, Materials, and Methods or the metadata in Supplementary File S2). In the SiTDB we also incorporated information on the trade volume that occurred within the European Union including the United Kingdom (EU-28) between 2015 and August 2020. For the number of species per category in the SiTDB see Table 3. The SiTDB and all related references are available in Supplementary File S1.

**Table 3:** Overview of the Songbirds in Trade database (SiTDB) with number of species per variable and percentage relative to the total number of songbird species (6659). For more detail see Experimental Design, Materials, and Methods.

Variable	Number of species	%
Trade Interest	1978	29.7
Domestic Trade	1137	17.1
International Trade	986	14.8
Primary Source of Trade (wild-caught/captive-bred)	1551	23.3
Trade as contributing threat to populations	206	3.1
Perceived relative trade volume globally	1555	23.4
Perceived relative trade volume internationally	988	14.8
Volume of EU trade	914	13.7
Wild source entering EU Trade after 2006	842	12.6
EU captive breeding confirmed	912	13.7
Available wild source in EU 2020	913	13.7
Substantial uncoordinated ex situ breeding effort ongoing 2020	180	2.7
Difficulty of captive breeding	1568	23.5
Coordinated transparent ex-situ breeding program established 2020	78	1.2
Known trade routes	805	12.1
Known Affected Subspecies	154	2.3
Domestication and mutations	34	0.5

#### 1.2.4 Types of use

We provide summarised data on species use from the IUCN Red List “usetrade“ data section, which contains information on the type of purpose for international and domestic trade as well as subsistence use for 1598, 764 and 750 species, respectively. Lastly, we collected preliminary data on the use of songbird species in traditional medicine from two sources with a focus on African species. Data were taken from the CITES List of species traded for medicinal purposes, which lists the parts used for medicinal purposes for three passerine species. For a further 108 passerine species used in, and traded for, traditional medicine in Africa we listed the data from Williams et al. [3].

### 1.3 Extinction risk

**Fig. 2:**
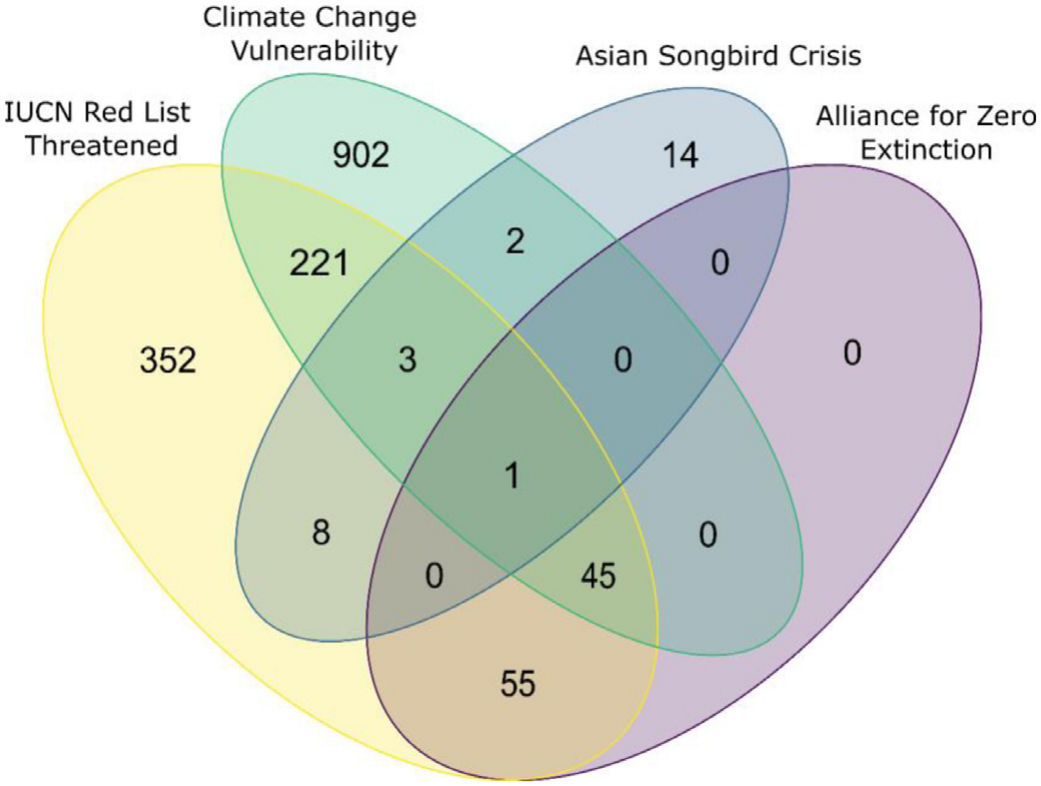
Venn diagram showing the overlap between the number of species that have been assessed as threatened (Vulnerable, Endangered, Critically Endangered) or Extinct in the Wild by the IUCN Red List (= Red List Threatened), highly vulnerable to climate change by Foden et al. (2013) (= High Vulnerability to Climate Change), highlighted by the IUCN SSC Asian Songbird Trade Specialist group as being part of the Asian Songbird Crisis (= Asian Songbird Crisis) and species listed as a Trigger Species by the Alliance for zero extinction (= Alliance for Zero extinction). The plot was made using the R package *VennDiagram* (Table 6).

In this knowledge area we included the threat status according to the IUCN Red List of Threatened Species (Version 2019-1). We also included the Alliance for Zero Extinction (AZE) trigger species, which are species listed as Endangered (EN) or Critically Endangered (CR) by the IUCN Red List, and for which over 95% of the resident population or one life history segment (such as breeding) exists in only one global site [4]. We provide the species’ International AZE site, the global population estimate and the percentage of the global population at the site according to AZE. We also provide the species vulnerability to climate change based on the IUCN Climate Change Vulnerability Assessment, as a climate change vulnerability score, which is either recorded as high (H) or low (L) for 5782 songbirds by Foden et al. 2013 [5]. Furthermore, we include the 28 songbird species identified at the Asian Songbird Crisis Summits in 2015 and 2017, listed as particularly threatened by the IUCN SSC Asian Songbird Trade Specialist Group [6]. The number of species in each of these schemes divided by their IUCN Red List category is shown in Table 4. The overlap of the number of species covered by the different risk schemes is shown in Fig. 2.

**Table 4:** Number of passerine species listed in different conservation prioritisation schemes per IUCN Red List Category including: Red List category, species with high vulnerability to climate change, Alliance for Zero Extinction (AZE) trigger species, and Asian Songbird Crisis Priority Species identified by the IUCN SSC Asian Songbird Trade Specialist Group. LC = Least Concern, NT = Near Threatened, VU = Vulnerable, EN = Endangered, CR = Critically Endangered, EW = Extinct in the Wild, EX = Extinct.

IUCN Red List		Total Number of Species		
	IUCN RL	High vulnerability	AZE Trigger	Asian Songbird
Category	category	to climate change	Species	Crisis
LC	5358	720	0	11
NT	527	173	0	5
VU	374	125	1	2
EN	216	105	49	5
CR	94	40	50	5
EW	1	0	1	0
EX	60	5	1	0
DD	29	6	0	0
**Total**	**6659**	**1174**	**102**	**28**

**Fig. 3:**
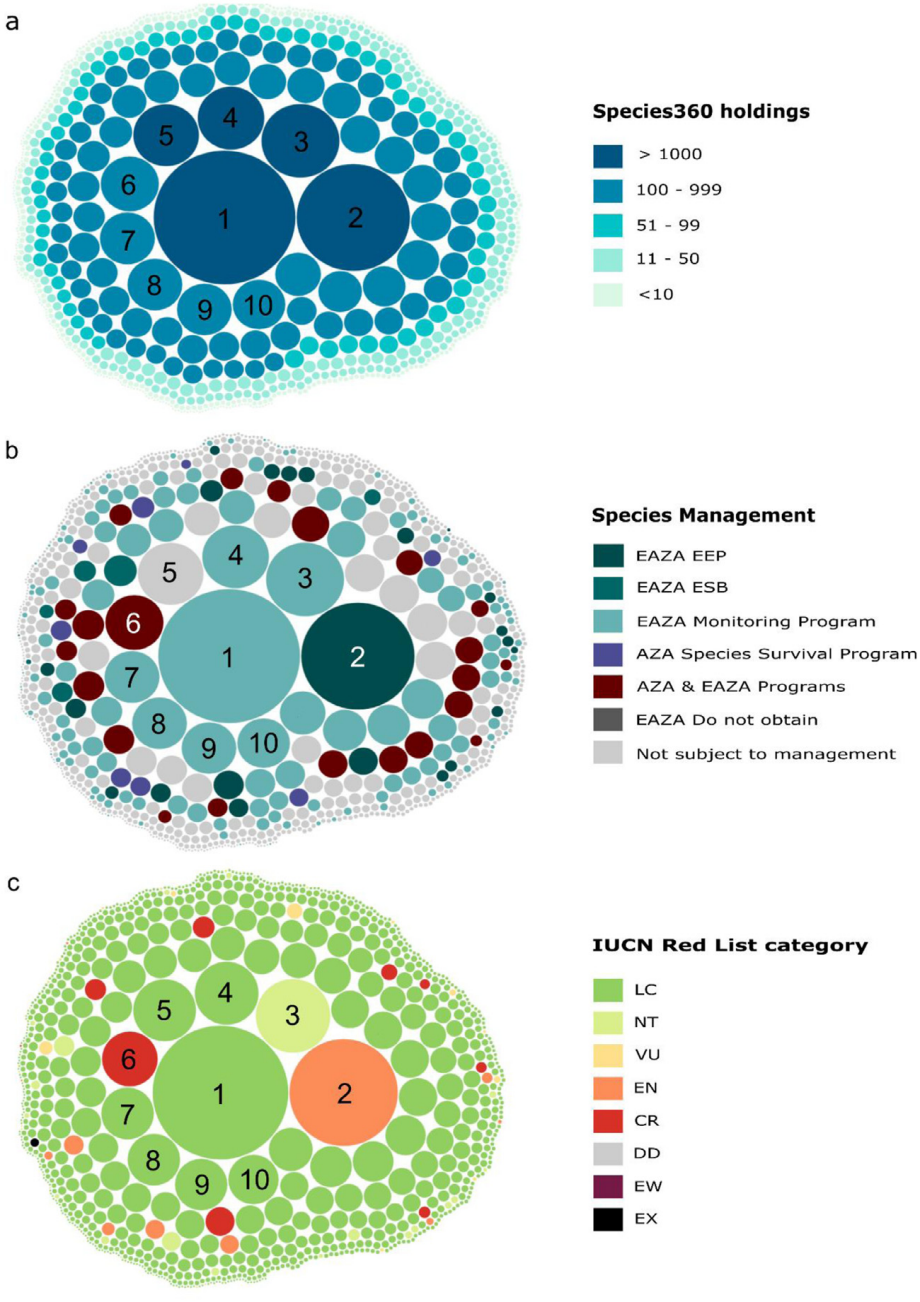
Circle chart showing the number of individuals per species kept in Species360 members (i.e., species holdings), their ex-situ management plans and IUCN Red List status. Each circle represents a species from the Species360/ZIMS database, and their circle size represents the number of individuals. The position of each species is the same across figures and the colour corresponds to either (a) the number of individuals, (b) the ex-situ management plan in the European Association for Zoos and Aquaria (EAZA) or the American Association for Zoos and Aquariums (AZA) or (c) their IUCN Red List status. Species with the 10 largest holding sizes are labelled: 1. *Taeniopygia guttata*, 2. *Lonchura oryzivora*, 3. *Chloebia gouldiae*, 4. *Ploceus cucullatus*, 5. *Ploceus castaneiceps*, 6. *Leucopsar rothschildi*, 7. *Foudia madagascariensis*, 8. *Quelea quelea*, 9. *Pycnonotus jocosus*, 10. *Lamprotornis superbus*. EEP = European Endangered Species Program, ESB = European Studbooks. LC = Least Concern, NT = Near Threatened, VU = Vulnerable, EN = Endangered, CR = Critically Endangered, EW = Extinct in the Wild, EX = Extinct. Plots were made using the R package *bubbles* (Table 6).

### 1.4 Management opportunities

The data recorded in this category contains information to evaluate species Conservation Opportunities for ex-situ management programs. Ex-situ programs include current and historical species holdings data from the Species360 network of more than 1,200 member organizations including zoos, aquariums, rescue centres and sanctuaries [7]. In total, 45718 individuals of 892 passerine species are currently kept in Species360 institutions (Fig. 3a) and 1910 species have been kept historically with the first record dating back to 1873.

We also included the total number of captive births per species at Species360 member organizations and the year in which a species was first kept historically. We also list whether a species is part of a regional species management plan, including members of the European Association of Zoos and Aquaria (EAZA) and the Association for Zoos and Aquariums (AZA) programs (Fig. 3b). Both EAZA and AZA have *regional species management plans* established with the aim to ensure that a species’ population across different zoo organizations is demographically and genetically sustainable. These plans have clear targets such as increasing a species’ population growth or maintaining a steady population structure and are usually managed across different institutions with clear record keeping of each individual's origin, pedigree and other husbandry, across which data is shared and coordinated, usually through a studbook keeper.

We include EAZA Regional Collection Plans (RCPs) which are divided into different levels of management intensity in the columns “EAZA_RCP_19”, “EAZA_RCP_comb”. The latter contains the EAZA RCPs that were updated in 2019 only for Southeast Asian songbirds as well as the still valid RCPs from 2017. For the RCP columns we have the following five categories: i) to indicate if the species is in a European Endangered Species Program “EEP”, ii) European Studbook “ESB”, iii) Monitored breeding program by assigned person “MON-P”, iv) Monitoring Breeding program by a Taxon Advisory Group “MON-T”, or v) if the species is listed as “do not obtain” (DNO) [8,9]. As of 2019, including those species in EAZA RCPs listed in 2017, there are a total of 244 recorded management plans for EAZA, 47 breeding programs and 194 monitoring plans, with 19 species under a MON-P and 178 under a MON-T and one species is listed as DNO. EEPs refers to species that have the highest management intensity, with a dedicated Species Committee that oversees demographic and genetic management to ensure the sustainability of the populations with clear targets (i.e. population growth or a steady population structure and genetic variability) usually managed through a studbook between the partners. ESBs have a slightly lower management intensity, although they keep a studbook with demographic and genetic analysis, breeding and transfer recommendations are not mandatory but encouraged. Additionally, the rules for non-EAZA member participation are less strict. MON-Ps have a lower management intensity with only a basic studbook, and species under a MON-T only have their population trend monitored by the Taxon Advisory Group. DNO species are not currently held by EAZA organizations and are not recommended to be obtained as they may be relevant taxa for conservation, education and communication purposes. We also include information in the SiTDB on ex-situ breeding programmes that are not managed by any regional Zoo or Aquarium Association (e.g., from commercial facilities), and those managed in regional breeding programs additionally to EAZA and AZA as well as the species’ difficulty of captive breeding based on personal observation and relevant literature.

### 1.5 Biological information

Here we present data on available biological information for each species regarding their ecology, demography, genomics and genetics (Table 5). The dataset contains information on body mass, clutch size and diet from Cooke et al. 2019 [10]. We also downloaded data on habitat (including upper and lower elevation limits, number of locations and habitat system), population size, generation length, movement patterns, number of subpopulations and distribution country, region and continent from the IUCN Red List website. The raw data can be downloaded directly from the IUCN Red List website. Here we only report whether the data was available or not. We included the life history data provided in the Demographic Species Knowledge Index (DSKI) database, including age of first reproduction, inter birth interval, clutch size, maximum recorded lifespan, proportion of reproductive females and recruitment, as well as the availability of crude mortality, population matrices or life tables available in Supplementary File S1. The data are presented as summarised information and in the original format. The final DSKI for mortality and fertility is also given, which is a composite measure of the level of available demographic data for each species [11]. We downloaded the species migratory status from the Global Register of Migratory Species (GROMS) website and their occurrence data from the Global Biodiversity Information Facility (GBIF) Occurrence Download.

**Table 5:** Summary of available biological data collected from eight data repositories, the Demographic Species Knowledge Index (Conde et al. 2019), the IUCN Red List, Cooke et al. 2018, the Global Register of Migratory Species (GROMS), the Global Biodiversity Information Facility (GBIF), the Bird 10 000 Genomes (B10K) Project, the Vertebrate Genome Project (VGP), GenBank, the Global Invasive Species Database (GISD) and the European Alien Species Information Network (EASIN).

Category	Number of species	%	Description
Body Mass	5850	87.9	Median adult body mass
Clutch size	Cooke: 2911 DSKI: 3518 Combined: 3618	43.7 52.9 54.4	Clutch size from Cooke et al. 2019, Number of eggs per female known in DSKI, Combined amount of information on clutch size available from DSKI and Cooke et al. 2019
Diet	5850	87.9	Diet type divided into 5 categories (1 = plant/seed, 2 = fruit/nectar, 3 = vertebrates, including carrion, 4 = invertebrates and 5 = omnivore)
Extent of Occurrence	6606	99.2	Extent of species occurrence
Elevation lower limit	2153	32.3	Upper limit of species occurrence
Elevation upper limit	4171	62.6	Lower limit of species occurrence
Population Size	1043	15.7	Population size range
Year Of Population Estimate	1576	23.7	Year population size was estimated
Locations Number	647	9.7	Number of locations species occurs at
Generation Length	6619	99.4	Length of one generation in years
Movement Pattern	6651	99.9	Movement patterns, Full Migrant, Altitudinal Migrant, Nomadic, Not a Migrant, Unknown
Subpopulation Numbers	700	10.5	Number of global subpopulations
Distribution country	6659	100	Countries in which species occurs
Distribution region	6658	99.9	Regions of species distribution
Distribution continent	6659	100	Continents of species distribution
Age at first reproduction	416	6.2	Age at first reproduction, for either one sex or unspecified sex
Crude mortality	427	6.4	Any information regarding crude death rates
Broods	622	9.3	Number of broods per year
Interbirth interval	19	0.3	Time between births in years
Life table	5	0.1	Life table with age or stage specific fertility and death rates available
Matrix death rates	115	1.7	Matrix with age or stage specific death rates available
Matrix fertility and death	86	1.3	Matrix with fertility and death rates available
Maximum recorded lifespan	586	8.8	Longest lifespan, time of individual carrying ring or maximum longevity
Proportion of reproductive females	11	0.2	Proportion of reproductive females per age class
Recruitment	1	0.02	Proportion of fledglings recruited as breeders in the local population
DSKI mortality	758	11.4	Index indicating quality of mortality data and availability across 22 data repositories standardized in Conde et al. 2019
DSKI fertility	3523	52.9	Index indicating quality of fertility data and availability across 22 data repositories standardized in Conde et al. 2019
DSKI mortality fertility	3540	53.2	Index indicating quality of mortality and fertility data and availability across 22 data repositories standardized in Conde et al. 2019
Migration GROMS	980	14.7	Migratory species according to the GROMS database
Number of Occurrences	6114	91.8	Total number of Occurrences recorded for each species in GBIF between 2000 and 2019, for all bases of record
Number of Occurrences from Observations	6095	91.5	Total number of Occurrences recorded for each species in GBIF between 2000 and 2019, with basis of record being either Observation, Human Observation or Machine Observation
B10K database	962	14.4	Species listed in the B10K database
VGP status	3	0.05	Status listed by the VGP
GenBank sequence type	4990	74.9	Number of species
Invasive Species GISD	15	0.2	Invasive species in the GISD
Alien Species In EU	140	2.1	Alien species not native in any part of the EU
IAS of Union Concern	2	0.03	Alien species of Union concern in the EU

**Fig. 4:**
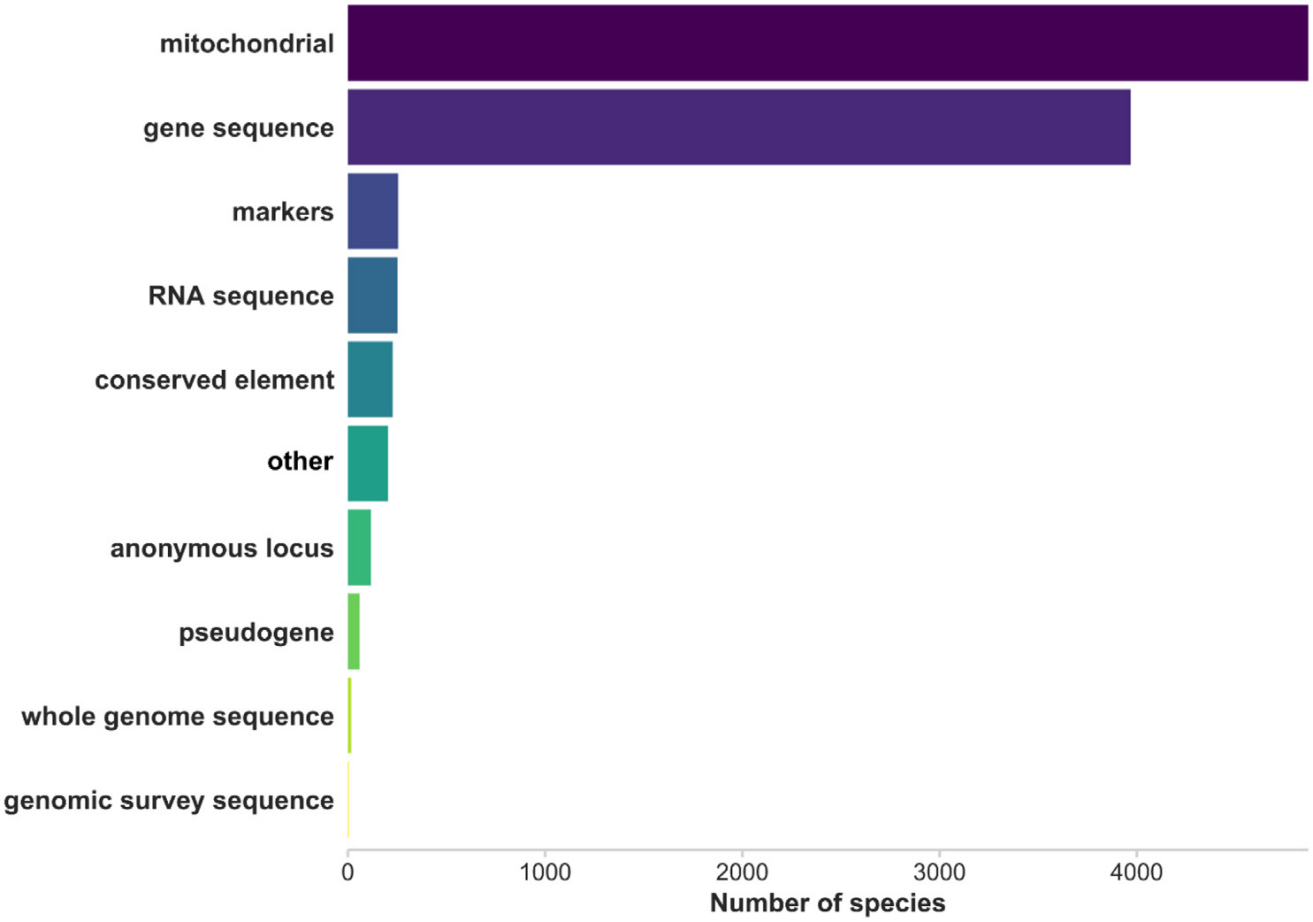
Number of species recorded on GenBank with a mitochondrial sequence (mitochondrial) gene sequence, genetic markers (markers), RNA sequence, conserved element, other type of genetic information (other), anonymous locus, pseudogene, whole genome sequence or genomic survey sequence. Plot was made in R using the *ggplot2* package (Table 6).

We provide the total number of occurrences per species from the Global Biodiversity Facility (GBIF) for all occurrence types for the years 2000 – 2019. In addition, we provide the number of occurrences based on only “human observation”, “machine observations” or “observations” for the same timeframe, to differentiate from other types of information such as origin of specimens in museum collections. Regarding genetic information, we list if data are available from the Bird 10,000 Genomes (B10K) project, the Vertebrate Genomes Project (VGP) and GenBank. The B10K and VGP status of sequencing the genomes are listed. For the B10K project the B10K ID, project phase, sample availability and appearance in their database are also specified. Data on the type of genetic sequences recorded on GenBank is recorded as gene sequence, mitochondrial sequence, RNA sequence, whole genome sequence, genetic markers, anonymous locus, other type of genetic information, genomic survey sequence, conserved element or pseudogene. Additionally, the number of records for each sequence type is indicated (Fig. 4).

**Fig. 5:**
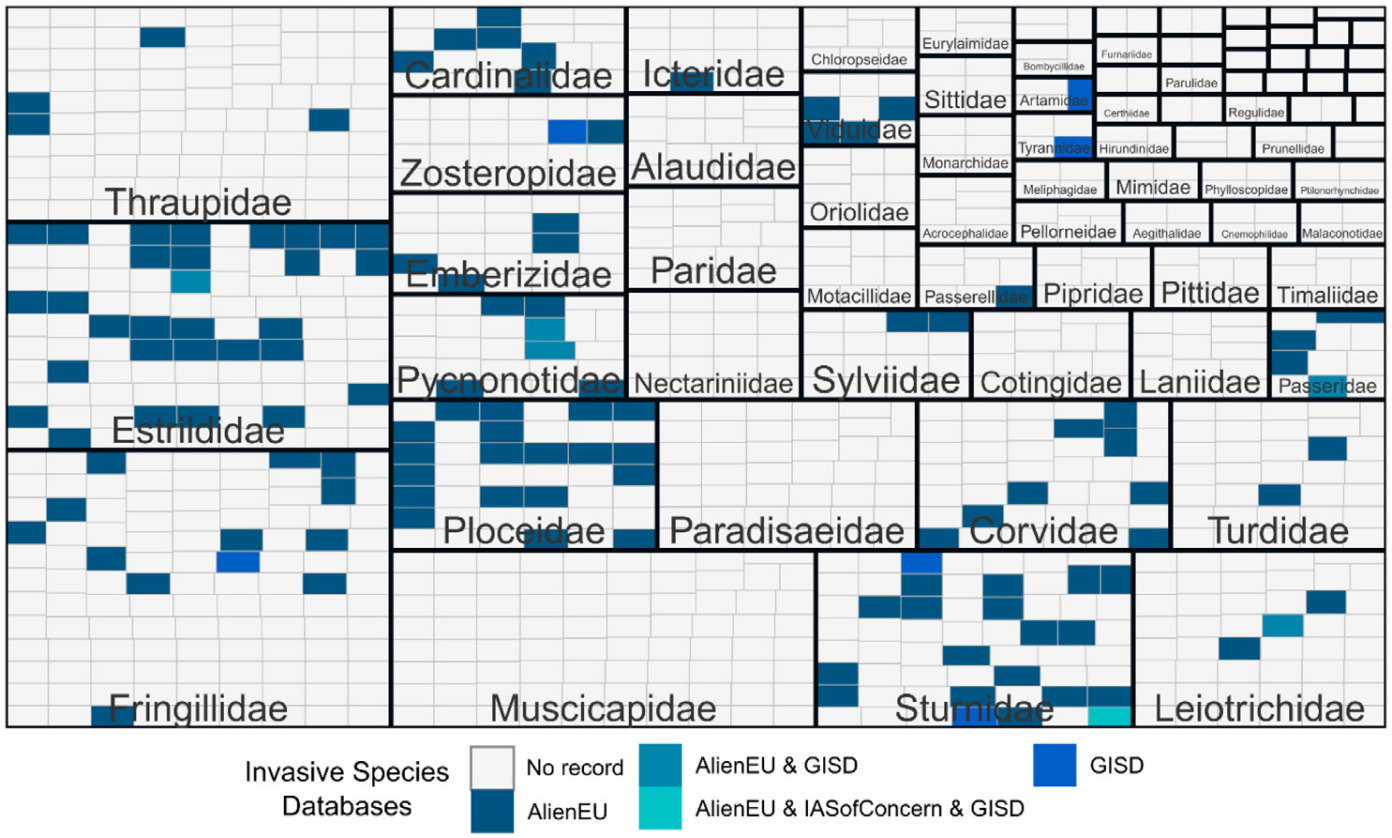
Treemap of species recorded in the Songbirds in Trade Database (SiTDB) as internationally traded and their listings in different Invasive Species databases. Each small square represents a species, ordered by families (bigger squares), coloured by their presence in the different databases. If species are covered by more than one database this is shown with a separate colour. AlienEU = Species recorded in the European Alien Species Information Network (EASIN) as alien in the EU, GISD = Species recorded in the Global Invasive Species Database, IASofConcern = Species recorded in the EASIN as alien and of Union concern. The plot was made using the R packages *treemapify* and *ggplot2* (Table 6).

We list the 15 songbird species recorded in the Global Invasive Species Database maintained by the Invasive Species Specialist Group of the IUCN. Furthermore, we obtained data from the European Alien Species Information network (EASIN) on alien and invasive songbird species in the EU with 140 and 2 species, respectively. Thus, data on species invasiveness, although limited, is also available (Fig. 5).

### 1.6 Intrinsic values

Data in this category are related to the intrinsic value of a species. For these measures, we collected information on evolutionary and ecological distinctiveness [12,13]. Evolutionary distinctiveness (ED) is a measure of the distance along the tree of life from one species to its next living relative and is given for 6587 species. A species which scores highly on ED and is also globally endangered (GE) based on the IUCN Red List is defined as an EDGE species by the Edge of Existence Programme [13]. Both, the evolutionary distinctiveness (ED) score, the globally endangered (GE) score and the combined EDGE rank are provided. Ecological distinctiveness assigns a value to a species’ distinct trait combinations and specialized ecological strategies and is listed for 6587 species. It is given as the mean with standard deviation, minimum, and maximum value in this dataset.

## 2 Experimental Design, Materials and Methods

The data we present are a compilation of 32 different data repositories (Table 1) including the SiTDB, which contains a diverse set of data including expert knowledge and species observed in market visits, explained in Section 2.2.3. To standardize across different data repositories, we used the Handbook of the Birds of the World and BirdLife International Digital Checklist of the Birds of the World Version 4. (December 2019) [2] as our backbone taxonomy by collating synonyms from the HBW/BirdLife checklist and a synonym list kindly provided by D. Lepage from Avibase.org [14]. When we did not find a species, we manually checked for their HBW/BirdLife synonym using Avibase.org. In a few cases where the name could not be found, mostly due to misspelling, we either used the Google search function to obtain the correct name or in case of ambiguities we validated the correct name based on expert knowledge. The final list contains 11027 names for the 6659 HBW/BirdLife species including the 6659 accepted names by HBW/Birdlife and 4349 synonyms for 2814 HBW/BirdLife species with synonyms (the synonym list is available in Supplementary File S3). We excluded 16 species because they were either not recognized by HBW/BirdLife, could not be matched to an HBW/BirdLife species, or had data entry errors. We did not perform a taxonomic standardization for subspecies present in several datasets but instead transferred all information to the relevant parent species, which was then standardized according to the HBW/Birdlife taxonomy. The only exception to this is the CITES listings data where subspecies listings were transferred to the accepted species name (e.g., *Gracula religiosa robusta* was assigned to *Gracula robusta,* the accepted HBW/Birdlife name and the relevant parent species after a recent taxonomical split and not to *Gracula religiosa* the previous parent species). To account for data differences between several species that were merged into one by the taxonomic standardization, we used either summed or mean values in case of numerical variables (e.g., we summed the total number of individuals in zoos for all species with the same HBW/Birdlife name). In the case of ranked categorical values, we used the higher value (e.g., DSKI). For the data on life history traits from DSKI we provide the full information including the data sources. All data were processed using R version 4.0.2 [15]. R packages used for both data collection and visualisation can be found in Table 6. The methods for data collection and cleaning are further described below for each knowledge area.

**Table 6:** List of R packages used for data collection and figures in this publication.

Package name	URL	Citation
rredlist	https://CRAN.R-project.org/package=rredlist	Scott Chamberlain (2020). rredlist: "IUCN" Red List Client. R package version 0.7.0.
countrycode	https://cran.r-project.org/web/packages/countrycode/index.html	Arel-Bundock et al., (2018). countrycode: An R package to convert country names and country codes. Journal of Open-Source Software, 3(28), 848. https://doi.org/10.21105/joss.00848
lemis	https://github.com/ecohealthalliance/lemis#readme, https://github.com/ecohealthalliance/lemis/tree/master/data-raw#readme.	Noam Ross, Evan A. Eskew, Allison M. White and Carlos Zambrana-Torrelio (2020). lemis: The LEMIS Wildlife Trade Database.
treemapify	https://CRAN.R-project.org/package=treemapify	David Wilkins (2019). treemapify: Draw Treemaps in "ggplot2". R package version 2.5.3.
bubbles	https://www.rdocumentation.org/packages/bubbles/versions/0.2	Joe Cheng, Mike Bostock and Jeff Heer (2020). bubbles: d3 Bubble Chart htmlwidget. R package version 0.2.
VennDiagram	https://CRAN.R-project.org/package=VennDiagram	Hanbo Chen (2018). VennDiagram: Generate High-Resolution Venn and Euler Plots. R package version 1.6.20.
taxize	https://f1000research.com/articles/2-191/v2 https://github.com/ropensci/taxize	Scott Chamberlain and Eduard Szocs (2013). taxize - taxonomic search and retrieval in R. F1000Research, 2:191. Scott Chamberlain, Eduard Szoecs, Zachary Foster, Zebulun Arendsee, Carl Boettiger, Karthik Ram, Ignasi Bartomeus, John Baumgartner, James O"Donnell, Jari Oksanen, Bastian Greshake Tzovaras, Philippe Marchand, Vinh Tran, Maëlle Salmon, Gaopeng Li, and Matthias Grenié. (2020) taxize: Taxonomic information from around the web. R package version 0.9.98.
ggplot2	https://cran.r-project.org/web/packages/ggplot2/index.html	H. Wickham. ggplot2: Elegant Graphics for Data Analysis. Springer-Verlag New York, 2016.

### 2.1 Conventions and treaties

Listings for CITES, CMS and the EU Wildlife Trade Regulations were downloaded from the Species+ website https://www.speciesplus.net/ operated by the United Nations Environment Program (UNEP) [16]. Information on species listing in the EU Bird Directive Annexes were obtained from the List of birds of the European Union on the European Commission website https://ec.europa.eu/environment/nature/conservation/wildbirds/eu_species/index_en.htm.

### 2.2 Human use

#### 2.2.1 Regulated trade

We obtained data on international trade from the annual reports submitted by CITES Parties from the CITES Trade Database (CITES, 2020) from 1975 to 2018 for all Passeriformes. We did not include the year 2019 as it was incomplete at the time of download (2020-10-08). We only included exports and imports in the dataset and excluded re-exports to avoid double counting trade transactions. We defined re-exports as all trades where the origin country differed from the exporting country or was reported as “XX” (i.e., unknown, rather than left blank). We summarized the total number of live individuals per species traded for commercial purposes (purpose code T) for each source for both the importer reported quantities and the exporter reported quantities. The data are presented for the two time frames 1975-2005 and 2006-2018 in order to make it comparable with the SiTDB, which is limited to data from 2006 onward.

Additionally, we downloaded data on wildlife and wildlife product imports into the United States from LEMIS curated by the EcoHealth Alliance using the R package *lemis* (Table 6). We only included records of transactions from live animals, or animals that died during transport (i.e. codes LIV or DEA in the “description” column), or other purposes than commercial trade (purpose code T). Only data entries with the unit “Number of Specimens” (code NO) were kept. We excluded potential data entry errors where live individuals were entered with the unit “kg”. We further excluded records referring to CITES-listed species (10 species), which made up less than 0.5% of the live animals in commercial trade. This was to avoid double counting, as these records are likely already included in the CITES Trade Database.

#### 2.2.2 Seizures

We obtained data on songbird seizures from the Wildlife Trade Information System compiled by TRAFFIC. For the purpose of this analysis, we only included seizures of whole individuals (i.e., commodity type = “Individual”, recorded as either live, dead or unknown), removing 51 observations that included eggs, meat, nests, skin, tails, plant parts, horns and 36 unknown commodity types. We also excluded 46459 individuals that were not identified to species or subspecies level (i.e., excluding genus, family or order level entries). One observation of the species *Prinia polychroa* was converted from weight into an estimated number of individuals based on the median bodyweight from Cooke et al. 2019 [10] (i.e., 38 kg/0.010975 kg = 3508 individuals). We manually checked all observations for data entry errors and duplicate entries and supplemented missing information in the columns “count”, “commodity type”, and “accumulated count” based on the descriptions of the seizure incidents. Some incident reports only mention rough estimates of the number of birds seized, such as “hundreds of birds” or “over 100 birds.” In these cases, we used the minimum possible amount, i.e., 200 individuals for “hundreds” and 100 birds for “over 100 birds”. We excluded records that did not mention any quantities such as "wild animals" or "birds". Some counts were recorded as "accumulated counts". These counts refer to an accumulated count of multiple commodities across multiple species where it was not possible to find the proportion of each species involved. For example, 45 birds were seized in one incident in Brazil (2018) and the seizure included four species and one genus. In these cases, we assumed an equal distribution of the amount across the different species or higher taxonomies identified. For example, in this case we divided the total count of 45 individuals by the total number of species (five), resulting in a count of 9 individuals per species. The database also notes for each record whether the count is based on an estimate or whether it refers to the actual amount. To calculate the total amount, we summed the estimated and the actual amount. Due to the accumulated counts, we sometimes obtained decimal values (such as 1.5 individuals). In those cases, we rounded to whole individuals. The data includes the location of the seizure and, in most cases, the locations of the trade route. Based on this information we divided the seizures into domestic and international trade as well as into trade with unknown trade destination. Some cases, however, did not report information on locations outside the seizure location, but international trade could potentially be inferred based on the nature of the incident (e.g., at an international airport or port) or species (e.g., non-native). However, for the purpose of this study we did not do a detailed analysis of this, potentially underestimating the number of incidents involving international trade. A list of species names confiscated or seized from 2006 to 2018 in the World WISE database was provided to us by the UNODC.

#### 2.2.3 Songbirds in trade database

We created the Songbirds in Trade Database (SiTDB) as a standalone data repository to include a diversity of information not present in global databases. It is constantly being updated, led and curated by S. Bruslund. In this paper we used Version 20200928-V1 from September 2020. The latest version is available from co-author S.Bruslund on request. The SiTDB only includes data on the trade of live individuals, thus information on derivatives such as meat, feathers, and samples are excluded. We include the following five types of data sources: i) market surveys, ii) records opportunistically captured from avicultural magazines and websites (i.e., sales advertisements and hobbyist breeders), iii) peer-reviewed literature, and iv) social media sale advertisements, and v) published and unpublished notes provided by experts. In the case of rarely traded species or species introduced to the trade more recently, close monitoring of social media proved useful, and provided considerable insights into poolry regulated trade. From the 6659 Passeriformes species, the SiTDB (Version 20200928-V1) identifies a total of 1589, of which 986 and 1137 species in international and domestic trade, respectively. In addition, we added records of the Wangi-wangi White-eye *Zosterops sp. novum,* a species recently discovered O'Connell et al. [17] and not yet described. Of the 1589 species, 466 (29%) have only one reference providing evidence of trade. The SiTDB incorporates and standardizes data since the year 2006 to track more recent trade trends including the effects of the EU import ban on wild birds [18]. We included information from 256 sources in nine languages (i.e., Dutch, English, French, German, Indonesian, Portuguese, Scandinavian, Spanish, Thai) including records from three published databases, 44 entries of expert knowledge, a legislation paper, 92 records from peer-reviewed literature (i.e., thesis, books or papers), 36 records from popular literature (articles, websites, press), and 77 entries based on social media (Facebook, WhatsApp Groups, Instagram). These references are supported with more than 400 data files (images and documents) available on request as indicated in Supplemental File S1. In addition, the SiTDB contains information on ex-situ management (see sections 7.-10. below). It is organized in the following sections:

1. *Trade Interest:* includes species for which live individuals are obtained or bred for personal or commercial use in the international and domestic trade, defined under the “SiTDB_trade_interest” column in S1, with two categories: “evidence” and possible trade “possible”. The category of “evidence” indicates those species for which we found references across the five different sources types described above, for species with confirmed trade interest documented by at least a single source. Species under the category of “possible” are those for which we found records before 2006 or species which are very popular or attractive in colour or voice, either obtained from personal communications for which we did not find confirmations, or evidence across the five data sources used.
2. *Domestic and International Trade:* indicates if we found evidence of trade either in the domestic or international markets, in the two separate columns “SiTDB_domestic_trade” and “SiTDB_international_trade” in table S1. Note that species traded internationally are usually also traded domestically before entering the international trade. However, in many cases it is difficult to document the domestic supply-chain trade leading up to the international trade component since these transactions are poorly regulated. Confirmed evidence is indicated with “yes”.
3. *Primary Source ofTrade:* indicates if more than 50% of the individuals traded were reported to be either wild caught or captive bred with the categories “wild caught”, “captive” or “unknown” respectively. Only in one case the primary source is indicated as “unknown”.
4. *Relative trade volume in relation to population size including both international and dome- stic trade between 2015-2020*: in column “SiTDB_ int_dom_perc_trade_vol_2015_20” we include a qualitative estimate of the combined volume of international and domestic trade relative to the species population size as reported by BirdLife International Data Zone [19], and classified it as: Low, Moderate, High, Extreme and Unknown (Table 7). These four volume categories were derived from the number of sources that reported a species being traded, in addition to the cases where the volume was quantified (i.e., in peer-review and seizure open data).

**Table 7:** Description of categorical variables in the columns “SiTDB_int_dom_perc_trade_vol_2015_20”, “SiTDB_int_perc_trade_vol_2015_21” and “SITDB_ vol_trade_in_eu_2015_20”. All perceived trade volumes are given relative to the population size.

Categorical variable	Description
Extreme	Indicates species for which the documentation of trade is recurring and continuously, or with trade numbers in thousands of individuals
High	Indicates species for which the documentation of trade is recurring and frequent, including species continuously found or volumes, in the hundreds of individuals, reported in trade across the five types of data sources. It accounts as well for proportionally high trade in threatened species with very small populations. For example, trade of 10 individuals can be considered high for a species such as the critically endangered Javan Green Magpie *Cissa thalassina* relative to its population size, estimated around 50 to 249 living individuals
Moderate	Indicates that we found trade recurring on a regular basis also applies for those species for which we found only few individuals being traded
Low	Indicates that a species is traded but appears only in one or few publications or data sources or only one or a few individuals are reported in the trade
Unknown	Indicates species for which we found evidence of trade but were not able to do a qualitative assessment of the level of trade given the data.
5. *Relative international trade volume in relation to population size between 2015-2020*: in column “SiTDB_int_perc_trade_vol_2015_21” as described above, here we include a qualitative estimate of the volume relative to a species’ population size as reported by BirdLife International Data Zone [19], but only for those records identified from the international trade. Following the same categorization explained in 4. and Table 7.
6. *Trade as a threat topopulations:* indicates if the trade (either international or domestic) is affecting the sustainability of a species or particular populations, only when we found this to be reported in the peer-review literature as “yes” in column “SiTDB_trade_as_threat”. Furthermore, we categorized species as “plausible” when a species is globally threatened according to the IUCN Red List and are traded with volumes in the categories of “High” or “Extreme” (Table 7). However, for species with range-restricted or small populations according to the BirdLife International Data Zone [19], we list them as “plausible” even if the trade volumes were in the categories of low or moderate.
7. *Difficulty of captive breeding*: in column “SiTDB_difficulty_breeding” we indicate if knowledge and technology to reliably breed the species is available based on expert knowledge. We indicate the level of breeding difficulty with the following categories: “challenging” for species with no or only accidental breeding success known, “hard” for species where breeding is possible in specialized settings and with considerable effort, “normal” for species found being bred consistently when good conditions are available, and “easy” for species identified to be bred routinely in captivity without much effort. This is a preliminary assessment based on personal observations of zoo employees, private breeders and literature such as avicultural magazines (included in the SiTDB source columns).
8. *Domestication and mutations*: in column “SiTDB_domestication_mutations” we indicate for every species with a “yes” whether regular visual domestication effect through altered phenotypes in size or colour is recorded, or if there is expert knowledge or popular publications. We recorded a total of 34 species, all of which are also in the international trade.
9. *Captivebreeding effort in the year 2020:* in column “SiTDB_breeding_effort_2020” we indicate as “yes” if the species is under a commercial, hobby or opportunistic breeding effort. These efforts have no coordination of breeding plans between organizations to ensure population sustainability and maximum long-term genetic diversity beyond personal needs of the respective breeder.
10. *Captive breedingprogramunder a regional species management plan in2020:* in column “SiTDB_breeding_program_2020” we indicate as “yes” species for which there are non-commercial breeding programs in place, which are usually governed by regional zoo associations but in some cases by governments or research entities. Usually, these programs have a species coordinator (i.e., studbookeeper) active in 2020 (see Supplementary File S1 for references). These include conservation breeding programs which are usually established as a result of an integrated planning process such as a regional collection plan in the case of zoos or a species action planning process with multiple stakeholders.
11. *Known trade routes:* In column “SiTDB_trade_routes” we recorded known trade routes when available based on the references cited in the SiTDB (S1). Since we did not conduct an in-depth analysis, this data may only cover only a limited geographical scope of the trade.
12. *Known subspecies in trade:* In column “SiTDB_affected_subspecies” we recorded subspecies in the trade if available from the literature, visits to markets, usually based on visual identification by experts (S. Bruslund, C. Shepherd, and B. Leupen). This provides additional information on the geographical scope of the trade and is useful also in allocating trade information in case of future taxonomic splits.

Furthermore the SiTDB contains a focused section on the songbird trade in the EU intended to aid EU policy makers and law enforcement:

13. *Trade in the European Union (EU 28) from 2015 to 2020*: in column “SITDB_vol_trade_in_eu_2015_20” we present a qualitative estimate of the volumes of trade within the EU (i.e., intra-EU trade) or imports into the EU, relative to a species’ population size as reported by BirdLife Data Zone [19], divided into the same categories of Low, Moderate, High and Extreme (Table 7). We did a qualitative categorization as explained above (2.2.3.4) considering only those records related to the EU.
14. C*aptive breeding confirmed in the EU:* Here we show if there are any records that indicate that a species has ever been bred in captivity within the EU. This is based on observations or publications (see types of data) and is given as “yes” to indicate recorded breeding or “unknown” in cases where documentation or empirical information is uncertain.
15. *Wild source entering EU Trade after 2006:* in column “SiTDB_wild_eu_after_06” we record indications of songbird trade into the EU (i.e., imports) from wild caught sources according to observations from visits to markets, social media and other types of trade advertisement. Likewise, we included those species that i) were found to be in advertisements for sale, ii) were not found to have been captive bred in the EU (i.e., column “SiTDB_eu_capt_breed” = “yes”), iii) or iv) are difficult to breed in captivity, thus column “SiTDB_difficulty_breeding” = hard or challenging. We indicate “yes” when conditions apply for a likely wild source entry.

#### 2.2.4 Types of use

Data on the use of species were collected from the IUCN Red List website https://www.iucnredlist.org/ using their Advanced Search function (IUCN Red List Version 2019-1). These data include trade types (i.e., food, pets & horticulture) and whether a species is traded internationally, domestically or used for subsistence. We summarised the information available per species and counted the number of purposes a species is used for separately for international and domestic trade and subsistence use. Information on a species’ use in traditional medicines was taken from CITES document AC18 Doc. 13.1 as well as Williams et al. [20,21]. Data from the CITES document includes which parts of the animal are used. The data are also presented as a combined list of species used in traditional medicine.

### 2.3 Extinction risk

The IUCN Red List status was taken from the Handbook of the Birds of the World and BirdLife checklist [1] which contains the latest updates from 2019. Data for the Alliance for Zero Extinction (AZE) trigger species was downloaded from the 2018 Global AZE Map at https://zeroextinction.org/site-identification/2018-global-aze-map/. Vulnerability to climate change assessments were retrieved from the supplementary data from Foden et al. 2013 [5]. Only the final climate change vulnerability score is listed in Supplementary File S1. Information on species involved in the Asian Songbird Crisis is based on Lee et al. 2016. All sources to obtain Extinction Risk data are listed in Table 1.

### 2.4 Management opportunities

We obtained zoo holdings data from the Species360 Zoological Information Management System (ZIMS), including globally shared data from over 1,200 zoos and aquariums worldwide following a data research request. The data contain the total number of individuals currently kept by Species360 members given as total counts and counts per sex, either recorded individually or in groups. We also calculated how many species are represented for each genus in the ZIMS holdings. Historical data on the number of captive births and the year of the first time a species was kept were also included [7]. Specific ex-situ management plans were obtained for two regional zoo associations. The Regional Collection Plans (RCP) for EAZA were retrieved from two internal EAZA publications provided by S. Bruslund and combined to obtain the full management list as of 2019 [8,9]. General notes on the management plans available in the 2019 publication are also listed in Supplementary File S1. Management plans for members of the American Association of Zoos and Aquariums (AZA), referred to as Species Survival Plans (SSP), were provided by M. Brauns (Program Assistant at AZA).

### 2.5 Biological information

Data on species diet, body mass and clutch size were taken from Cooke et al [10]. Information on extent as well as upper and lower elevation limits of occurrence, population size, year of population estimate, locations number, generation length, movement pattern and subpopulation number were downloaded from the IUCN Red List website https://www.iucnredlist.org/ using the advanced search and download functions. Distribution countries were downloaded using the *rredlist* package in R (Table 6). The countries were then converted to their respective regions and continents using the *countrycode* package in R (Table 6). Demographic data was collected from DSKI [11]. The data was summarised based on the demovar variables (“Age at first reproduction”, “Inter-litter/Inter-birth interval”, “Litter/Clutch size”, “Maximum recorded lifespan”, “Crude mortality”, “Matrix with age- or stage- specific death rates”, “Matrix with age- and stage-specific death rates”, “Matrix with age- and stage-specific fertility and death rates”, “Recruitment”, “Proportion of reproductive females” and “Lifetable with age- or stage-specific fertility and death rates”) and is given as mean and range in case of numerical variables. Categorical variables record the availability of data which is indicated with “yes”. In the case of the “Crude mortality” demovar the separate measures cannot be summarised therefore this variable is given as availability of data in the original database. Further information on the migratory status of species was downloaded from the website of GROMS http://groms.de/groms_neu/view/order_stat_patt_spanish.php?search_pattern=. Number of occurrences were collected from the GBIF website using their “Get data” tool. To get an overall impression on occurrence as well as live occurrences, the data were filtered in two ways. One set of data contains all occurrence data for Passeriformes from 2000 – 2019. The second dataset consists of occurrence data contrived only from the basis of record types “observation”, “machine observation” and “human observation” from 2000–2019. We downloaded the Bird 10 000 Genomes (B10K) Project data using the Species Search function on their website https://b10k.genomics.cn/species.html. The Vertebrate Genome Project status was downloaded from the website http://vgpdb.snu.ac.kr/details/ filtering for Passeriformes. Using the R package *taxize* we downloaded genomic information from GenBank (Table 6). The resulting text file containing descriptions of the sequence records was mined for words referring to record types. We split these types into the categories: “gene sequence”, “mitochondrial sequence”, “RNA sequence”, “whole genome sequence”, “markers”, “anonymous locus”, “genomic survey sequence”, “conserved element”, “pseudogene” and “other information”. The data from the Global Invasive Species Database was likewise downloaded from http://www.iucngisd.org/gisd/ after using the advanced search function to specify the order Passeriformes. Information on the alien and invasive species of the EU was obtained from the European Alien Species Information Network website https://easin.jrc.ec.europa.eu/easin. The data were filtered once for alien species not native in any part of the EU and of EU concern to get invasive species of EU concern and once for alien species not partly native in the EU to get all non-native alien species.

### 2.6 Intrinsic values

Evolutionary Distinctiveness (ED) scores, Globally Endangered (GE) scores and EDGE ranks were downloaded from the EDGE Lists at https://www.edgeofexistence.org/%20edge-lists/. Ecological Distinctiveness values were extracted from the supplementary material from Cooke et al. 2020 [12].

## CRediT Author Statement

**Dalia A. Conde:** Conceptualization, Supervision, Project administration, Writing, Funding acquisition; **Jacqueline Juergens:** Data Curation, Methodology, Formal analysis, Investigation, Writing – Original Draft, Funding acquisition; **Simon Bruslund:** Data Curation, Validation, Investigation, Methodology, Writing - Original Draft, SiTDB leadership and curator; **Johanna Staerk:** Data Curation, Methodology, Formal analysis, Investigation, Writing - Original Draft; **Rikke Oegelund Nielsen:** Formal analysis, Methodology, Data curation; **Chris Shepherd:** Investigation, Validation, SiTDB; **Boyd Leupen:** Investigation, Validation, SiTDB; **Kanitha Krishnasamy:** Investigation, TRAFFIC data; **Serene Chng:** Investigation, TRAFFIC data; **John Jackson:** Formal analysis, Methodology, Data curation; **Antony Bagott:** Investigation, TRAFFIC data; **Romulo Alves:** Data, Formal analysis, Investigation. All authors contributed to Writing - Review & Editing.

## Declaration of Competing Interest

The authors declare that they have no known competing financial interests or personal relationships which have, or could be perceived to have, influenced the work reported in this article.

## Appendix Supplementary materials

Image, application 1

Image, application 2

Image, application 3

## Footnotes

Supplementary material associated with this article can be found in the online version at doi:10.1016/j.dib.2021.107093.
